# Intraepithelial lesions of the uterine cervix during pregnancy: reaffirming the safety of conservative management

**DOI:** 10.1016/j.gore.2025.101792

**Published:** 2025-06-23

**Authors:** Meirovitz Mihai, Pasternak Maya, Kessous Roy, Davidesko Sharon

**Affiliations:** a Department of Obstetrics and Gynecology, Soroka University Medical Center, Ben-Gurion University of the Negev, Beer-Sheva, Israel; b Department of Gynecological Oncology, Soroka University Medical Center, Ben-Gurion University of the Negev, Beer-Sheva, Israel; c Faculty of Health Sciences, Ben Gurion University of the Negev, Beer Sheva, Israel

**Keywords:** Conservative management, Pregnancy, High-grade squamous intraepithelial lesion (HGSIL), Low-grade squamous intraepithelial lesion (LGSIL), Cervical intraepithelial neoplasia (CIN), Excision

## Abstract

**Objectives:**

Diagnosis of high-grade squamous intraepithelial lesions (HGSIL) during pregnancy is not uncommon, and managing these cases presents clinical challenges. In this study we evaluated the safety and outcomes of conservative management for high-grade squamous intraepithelial lesions (HGSIL) diagnosed during pregnancy.

**Methods:**

A retrospective case series was conducted at Soroka University Medical Center (2011–2020) including pregnant women with histologically confirmed HGSIL managed conservatively during pregnancy and treated postpartum.

**Results:**

Thirty-two women were included. The mean age at diagnosis was 31.2 ± 3.4 years, with most diagnosed in the first trimester (mean gestational age 8.1 ± 4.1 weeks). The average gestational age at delivery was 39.3 ± 1.0 weeks, and mean birth weight was 3045.6 ± 501.7 g. Postpartum conization showed complete regression in 15.6 %, regression to LGSIL in 6.3 %, and persistent HGSIL in 78.1 %. No cases progressed to invasive cancer. During 5.7 ± 2.9 years of follow-up, three patients (9.4%) experienced recurrence requiring repeat conization.

**Conclusions:**

Conservative management of HGSIL during pregnancy appears to be a safe and effective approach. In our case series, no progression to malignancy was observed, and postpartum outcomes were favourable. Deferring excisional treatment until after delivery is supported by these findings**.**

## Highlights

• Conservative management of cervical HGSIL in pregnancy shows low progression risk to invasive disease.
• Case series: 78 % HGSIL persistence, 22 % regression, zero progression with 5.7-year follow-up.
• In our 32-patient series, no progression to malignancy occurred with conservative management.
• Literature review supports low progression rates of HGSIL during pregnancy across multiple studies.

## Graphical abstract

**Figure:**
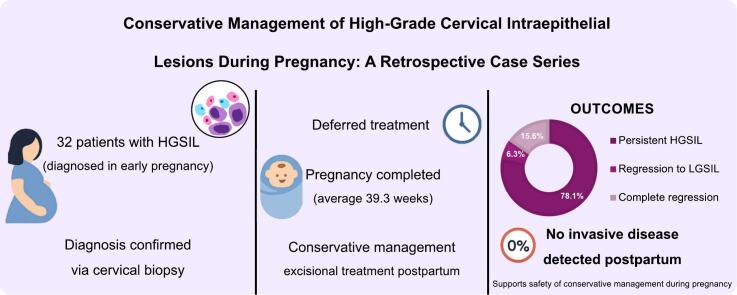

## 1 Introduction

Screening for cervical cancer enables the identification of pre-invasive lesions and timely intervention. The implementation of structured screening programs has contributed to a reduction of more than 50 % in both the incidence and mortality of cervical cancer over the past three decades (Cohen et al., 2019). High-grade squamous intraepithelial lesion (HGSIL) is caused by oncogenic strains of the human papillomavirus (HPV) that affect the transformation zone of the cervix (Bansal et al., 2008, Loopik et al., 2021, Kocken et al., 2011 May). HGSIL includes cervical intraepithelial neoplasia (CIN) 2 and 3, with CIN3 considered a pre-invasive lesion (Alrajjal et al., 2021, Egemen et al., 2020 Apr). The probability of CIN3 progressing to invasive cancer over 30 years is estimated to be approximately 10–30 %, with more recent studies suggesting rates closer to 10 % (Demeter et al., 2002). Current cervical cancer screening involves HPV testing and/or cytology. When screening results are abnormal, women are referred for colposcopy based on risk-stratification protocols (Beharee et al., 2019 Sep). If colposcopy findings are concerning, targeted biopsies are performed (Basu et al., 2018). The severity of the condition is evaluated through standardized histological criteria (Kamal, 2022). While low-grade squamous intraepithelial lesions (LGSIL) may be monitored using risk-based surveillance, HGSIL generally requires treatment such as large loop excision of the transformation zone (LLETZ) or cone biopsy. Various studies have suggested that LLETZ is preferred over cold knife conization (CKC) because of its lower risk of complications (Pina et al., 2013 Jun 20, Jiang et al., 2017). Treatment choice depends on the patient's age, medical history, and the extent and location of the affected region. Treatment also depends on the ability and experience of the clinical team. After treatment, patients are monitored through HPV testing and cytology, with colposcopy performed when indicated by post-treatment risk assessment protocols (Bansal et al., 2008, Loopik et al., 2021, Egemen et al., 2020 Apr, Davidesko et al., 2023). The most common acute complications observed following these procedures are bleeding, infections, and damage to surrounding organs (e.g., injury to the bladder or urethra) (Santesso et al., 2016 Mar). Women who undergo LLETZ/ CKC are also at an increased risk for long-term complications, including premature delivery (Kyrgiou et al., 2016 Jul, Kiuchi et al., 2016) and cervical stenosis (Condrat et al., 2021 Dec 7).

The highest rate of HPV colonization typically occurs during the reproductive years, and it is not uncommon for HPV-positive patients to be diagnosed during pregnancy (Condrat et al., 2021 Dec 7, Murta et al., 2002). Accordingly, cervical cancer is one of the more prevalent malignancies among pregnant women, with an estimated incidence of up to 1.5 cases per 10,000 pregnancies (Demeter et al., 2002, Beharee et al., 2019 Sep).

Typically, managing abnormal screening results during pregnancy follows a similar pathway as

nonpregnant patients, although the risks of diagnosis and treatment require special consideration regarding the timing of these procedures in this population. Physiological changes in the cervix during pregnancy may impede the identification of cancerous lesions through colposcopy and present a diagnostic challenge (Ciavattini et al., 2018 Oct). These changes include increased vascularity, hypertrophy of the cervical epithelium, and increased production of cervical mucus, which may obscure the visualization of abnormal tissue (Hunter et al., 2008 Jul). Therefore, HGSIL may not be accurately diagnosed during pregnancy. HGSIL lesions typically exhibit features such as abnormal glandular architecture, increased nuclear-to-cytoplasmic ratio, and abnormal mitoses (Arends et al., 1998 Feb). These features can be challenging to diagnose during pregnancy owing to the aforementioned physiological changes in the cervix. Generally, colposcopy with biopsy during pregnancy does not pose significant surgical or obstetric risks and clinically necessary biopsies can be conducted safely (Khan et al., 2017 Oct). There is a small risk of bleeding associated with cervical biopsy, which may be further increased during pregnancy due to the increased vascularity of the cervix; however, the risk of bleeding is relatively low when appropriate precautions are taken (Massad et al., 2013 Apr).

Routine screening for cervical cancer is not explicitly indicated in pregnant women, resulting in a relatively limited number of patients with abnormal cervical testing outcomes during pregnancy. Furthermore, the scarcity of complications and intricate ethical considerations surrounding research during pregnancy contribute to the paucity of numerical data on this matter. As a result, there is a lack of randomized controlled trials and extensive research investigating the potential complications associated with performing cervical biopsies during pregnancy.

While low-grade lesions can be managed with surveillance for the duration of pregnancy owing to the indolent nature of the disease (Bansal et al., 2008, Loopik et al., 2021), the diagnosis of HGSIL during pregnancy is associated with high levels of maternal stress, and the management of these cases remains debatable (Beharee et al., 2019 Sep, Murta et al., 2002). Questions regarding the ideal timing for cervical conization are still unclear, with the possibility that delaying conization to the postpartum period may pose a risk of progression to malignancy (Loopik et al., 2021, Kaplan et al., 2004 Aug 25, Muller and Smith, 2005 Dec). Conversely, performing the procedure during pregnancy exposes both the mother and fetus to potential complications (Demeter et al., 2002, Robinson et al., 1997 Jan, Gao et al., 2022, Penna et al., 1998). One of the most commonly reported complications of conization during pregnancy is the increased risk of preterm delivery, which may result in neonatal morbidity and mortality (Robinson et al., 1997 Jan). Another reported risk is potential cervical incompetence, which increases the likelihood of miscarriages or preterm births (Demeter et al., 2002, Robinson et al., 1997 Jan). Conization can also cause bleeding, which may require blood transfusion or other interventions (Robinson et al., 1997 Jan). Infection (Demeter et al., 2002, Gao et al., 2022) is another potential complication of conization. Additionally, conization may cause rupture of membranes, including preterm premature rupture of membranes (PPROM), which may progress to preterm labor (Gao et al., 2022). Fetal injury, including placental abruption, fetal bleeding, and stillbirth, is another possible but rare complication of conization (Demeter et al., 2002, Robinson et al., 1997 Jan, Penna et al., 1998).

Given the potential risks associated with both immediate intervention and delayed treatment, understanding the natural course of HGSIL during pregnancy is essential. Improving evidence in this area is crucial to guide clinical decision-making and provide appropriate counseling to women diagnosed with HGSIL while pregnant.

## 2 Methods

We conducted a retrospective case series analysis of patients diagnosed with HGSIL during pregnancy at Soroka University Medical Center between 2011 and 2020. Cases were identified through review of the institutional colposcopy database. Inclusion criteria were: (1) histologically confirmed HGSIL on cervical biopsy during pregnancy, (2) conservative management during pregnancy with excisional treatment deferred until postpartum, and (3) complete follow-up data including postpartum pathology results. We collected demographic data, risk factors, initial diagnostic test results, obstetric outcomes, postpartum pathology results, and follow-up data.

This study was conducted in accordance with institutional guidelines for retrospective data analysis.

### 2.1 Case series

In Table 1 we present a case series of 32 women diagnosed with HGSIL on cervical biopsy during pregnancy between 2011 and 2020 at Soroka University Medical Center, a tertiary referral center in Beer-Sheva, Israel. Women were referred for colposcopy in early pregnancy, and those with suspicious lesions underwent cervical punch biopsy to rule out invasive disease requiring urgent intervention. The mean age at diagnosis was 31.2 ± 3.4. Most women were diagnosed in the first trimester with a mean gestational age at diagnosis of 8.1 ± 4.1 weeks. Of the women included in the study, six were classified as obese (18.8 %), 16 were smokers (50 %), and eighteen women reported previous use of oral contraceptives (56.2 %). The initial diagnostic Pap smear demonstrated HGSIL in 18 patients (56.2 %), low-grade squamous intraepithelial lesion (LGSIL) in ten patients (31.2 %), atypical squamous cells HGSIL could not be excluded (ASC-H) in three (9.4 %), and no dysplasia was seen in one patient (3.1 %) who was referred for colposcopy testing due to high-risk HPV DNA. All patients demonstrated high-grade squamous intraepithelial lesions (HGSIL) on cervical biopsy. The average gestational age at delivery was 39.3 ± 1 weeks, with an average birth weight of 3045.6 ± 501.1 g. Seven patients (21.9 %) underwent cesarean delivery because of obstetric indications unrelated to the HGSIL diagnosis. After delivery, all the patients underwent conization as their definitive treatment, on average 53.1 ± 5 weeks after initial diagnosis. Of the 32 patients, five (15.6 %) had complete regression of the disease, two (6.2 %) showed regression to LGSIL, and the remaining 25 (78.1 %) had persistent HGSIL. None of the women in the study progressed to malignancy in the postpartum pathological evaluation. Eight patients (25 %) showed pathological involvement of the margins, and seven (21.9 %) had endocervical gland involvement. We performed a follow-up of 5.7 ± 2.9 years. Twenty-seven women (84.3 %) underwent a one-year post-conization smear, and 23 (71.9 %) underwent a test two or more years after conization. Of these women, two (6.2 %) demonstrated ASCUS cytology and one patient (3.1 %) was diagnosed with LGSIL. Three women (9.4 %) underwent repeat conization for a persistent disease.

**Table 1:** Characteristics and outcomes of the study population (*n* = 32).

**Demographic Characteristics and Risk Factors**		
Age (years)		31 ± 3
Obesity		6 (19 %)
Smoking		16 (53 %)
Previous oral contraceptive use		18 (56 %)

**Diagnostic tests**		
PAP	LGSIL	10 (31 %)
	ASC-H	3 (9 %)
	HGSIL	18 (56 %)
	No dysplasia	1 (3 %)

**Obstetric outcomes**		
Gestational age at diagnosis (weeks)		8.1 ± 4.1
Cesarean section		7 (22 %)
Gestational age at delivery (weeks)		39.3 ± 1
Birth weight (grams)		3045.6 ± 501.7

**Conization**		
Interval between diagnosis andtreatment (weeks)		53.1 ± 5
Endocervical gland involvement		7 (22 %)
Pathological involvement of margins		8 (25 %)
Pathology	No dysplasia	5 (16 %)
	CIN 1	2 (6 %)
	CIN 2–3	25 (78 %)

**Follow-up**		
Repeat conization		3 (9 %)
Duration of follow-up (years)		5.7 ± 2.9
Post-conization Pap	6 months	21 (66 %)
	1 year	27 (84 %)
	2 years or more	23 (72 %)
**Recurrence**		3 (9 %)
Cytology of recurrence	ASC-US	2 (67 %)
	LGSIL	1 (33 %)

*Data presented as n (%) or mean ± SD as appropriate.*

*Abbreviations:****ASC-H****– atypical squamous cells HGSIL cannot be excluded,****ASCUS****– atypical squamous cells of unknown significance,****CIN****– cervical intraepithelial neoplasia,****HGSIL****– high-grade squamous intraepithelial lesion,****LGSIL****– low-grade squamous intraepithelial lesion,****PAP****– Papanicolaou test*.

*all patients had HGSIL on cervical biopsy at diagnosis.

## 3 Discussion

The management of cervical HGSIL during pregnancy remains unclear, leading to varying management strategies based on clinician experience, ranging from conservative surveillance to active surgical intervention (conization). This study contributes to current knowledge by presenting a case series includes thirty-two HGSIL cases diagnosed during pregnancy with high compliance rates to follow-up after conization over a period that exceeded the average time for recurrence (Jakobsson et al., 2009 May).

An important finding in our series is the 22 % overall regression rate (15.6 % complete, 6.3 % to LGSIL) observed postpartum. This is clinically significant, suggesting that while HGSIL carries a long-term progression risk, many lesions remain stable or regress, particularly during pregnancy. Physiological changes in pregnancy, such as hormonal and immunologic shifts, may influence lesion behavior. Our data reinforce that pregnancy does not appear to accelerate malignant transformation. Given the risks of conization during pregnancy, including hemorrhage and preterm delivery, the observed regression rate strengthens the case for conservative management with postpartum re-evaluation.

Our findings are consistent with prior studies evaluating the natural progression of HGSIL during pregnancy. Freudenreich et al. (Freudenreich et al., 2022 Dec) reported a 35.6 % regression rate and a low progression rate of 1.5 % to malignancy in pregnant patients with CIN 2,3. Vlahos et al. (Vlahos et al., 2002) demonstrated a 61.5 % regression rate to low-grade lesions in pregnant women diagnosed with HGSIL, and no cases that progressed to malignancy. Other studies have reported regression rates ranging from approximately 28 % to over 60 % (Suchonska et al., 2020, Mailath-Pokorny et al., 2016 Dec 7, Siegler et al., 2014 Apr, Paraskevaidis et al., 2002 Aug, Yost et al., 1999 Mar), reinforcing that most high-grade lesions in pregnancy either remain stable or regress, with a very low likelihood of progression to invasive cancer. While our 22 % regression rate is somewhat lower than the highest rates reported, it remains aligned with the general trend and supports the safety of conservative management during pregnancy. It is important to note that some variability across studies may be attributed to differences in study design, sample size, diagnostic criteria, and follow-up protocols, factors that may influence the reported rates of regression and progression.

Our management strategy also aligns closely with current international guidelines. The American Society for Colposcopy and Cervical Pathology (ASCCP) and the Royal College of Obstetricians and Gynecologists (RCOG) recommend that all pregnant women with HGSIL undergo colposcopy-guided biopsy performed by a trained physician (Massad et al., 2013 Apr, Nelson, 2021, Royal College of Obstetricians and Gynaecologists, 2013). The ASCCP further recommends continued surveillance every 12–24 weeks through cytology and colposcopy, with deferral of excisional treatment unless invasive disease is suspected (Massad et al., 2013 Apr, Nelson, 2021). FIGO similarly advises avoiding treatment for CIN during pregnancy due to the associated risks and reserves excisional procedures for suspected invasive cancer (International Federation of Gynecology and Obstetrics (FIGO), 2009). In our series, no cases progressed to invasive disease, and all patients underwent excisional treatment postpartum, consistent with these guidelines and supporting their safety and efficacy in clinical practice.

Although some studies, such as that by Kaplan et al., reported a higher progression rate of 11 % to malignancy (Kaplan et al., 2004 Aug 25), the majority of available literature, including studies by Suchonska et al., Mailath-Pokorny et al., and Yost et al., corroborate our findings of low progression rates and high rates of postpartum regression (Freudenreich et al., 2022 Dec, Vlahos et al., 2002, Suchonska et al., 2020, Mailath-Pokorny et al., 2016 Dec 7, Siegler et al., 2014 Apr, Paraskevaidis et al., 2002 Aug, Yost et al., 1999 Mar). Collectively, this evidence reinforces that conservative management with careful surveillance remains a safe strategy, mitigating the risks associated with unnecessary surgical intervention during pregnancy.

The strengths of our study come from original data from our case series. The extended follow-up period led us to high certainty that all cases of recurrence were identified during the study period. We acknowledge limitations including the retrospective nature of our case series, which introduces a potential data collection and analysis bias. However, this was considered minimal in the present study due to the fact that clinical data was registered directly to a computerized medical registration system by trained physicians and reviewed by trained medical secretaries before final archiving hence, reducing the risk of errors and recall bias. Another limitation is the single-center design, which may limit the generalizability of our findings to other healthcare settings or populations.

Given our findings and the known risks of excisional procedures during pregnancy, conservative management with close surveillance appears to be a safe and effective strategy for HGSIL diagnosed in pregnancy. Deferring treatment until postpartum avoids unnecessary interventions and minimizes obstetric complications. Future prospective studies are warranted to better understand the biological mechanisms underlying lesion regression during pregnancy and to refine guidelines for optimal timing of intervention.

## Funding and Competing Interests

The authors declare that no funds, grants, or other support were received during the preparation of this manuscript, The authors have no relevant financial or non-financial interests to disclose.

## Declaration of competing interest

The authors declare that they have no known competing financial interests or personal relationships that could have appeared to influence the work reported in this paper.
